# Examination of the Pupils.—III

**Published:** 1909-04-24

**Authors:** 


### Medicine.

### EXAMINATION OF THE PUPILS.—III.

*The Outline of the Pupils.*—Another point that should receive attention is the shape of the pupil—as to whether it is round or oval, centric or excentric, regular or irregular in outline. Irregularity would suggest some affection of the iris itself; indeed, any abnormality in the shape of the pupils is chiefly of importance as an indication of local disease or malformation, rather than as a symptom of any nervous disease.

Finally, there are three contractile reactions of the pupils that should be tested—namely, to light, to convergence, and to painful stimulation of the skin of the neck.

*Dilatation on pinching the skin of the neck.*—This is by a long way the least important of the three. The normal pupil should dilate in response to a sharp nipping of the skin over the neck. Absence of such dilatation, however, is not necessarily pathological, and at most it could only point either to fixation of the iris, paralysis of the cervical sympathetic, or the action of some drug such as belladonna or eserine. Everything that can be learnt from the reflex can be learned, so far as present knowledge goes, without it.

*The reaction to light.*—When the light which is entering the eye becomes brighter the healthy pupil immediately contracts; it is a reflex act, and much can be learned from abnormalities in it. In the first place, however, it is important that the reaction should be tested for in a proper manner. To cover up both eyes with the hands and then to suddenly remove the latter and at the same time try to observe the pupillary changes is a very common but a very imperfect method of making the examination, and this for two reasons: in the first place, the reaction obtained in one eye may be due to light entering the other, a source of fallacy which can only be avoided by testing each eye separately; in the second place, the pupil often responds so rapidly that its movements cannot always be followed with certainty unless the pupil can be watched before exposure to the brighter light as well as at the moment of that exposure.

Under normal circumstances a beam of light thrown into one eye only causes both pupils to contract. If, therefore, steps are not taken to cover up the opposite eye, the pupil of the eye which is being examined will be as much contracted whilst it is shielded from the light as it will be when the light is allowed to fall on it again.

A very good way of testing the light reflex is as follows:—Supposing the right eye to have been closed by one of the observer's hands whilst the patient is sitting opposite a lamp or a bright window, the observer should interpose some opaque object, such as a book or his other hand, between the lamp or window and the eye that is being examined. The interposed object should be at such a distance from the patient's face that the pupil can be watched all the time. On removing the interposed object the full light from the lamp or the window will fall upon a pupil that has been watched whilst in the comparative dark, and one can then tell whether it contracts quickly or sluggishly or not at all. It is better to make use of light coming from a distance rather than of a match at close quarters, because not only may the warmth of the match cause the patient to start and his pupil consequently to move emotionally, but also it may be difficult to prevent the patient from looking directly at the match and thus causing contraction of the pupil from accommodation. If, however, the patient can fix his mind upon distance and learn to pay no attention to the lighted match, it is not a bad method of examining the light reflex to have the patient in a dim light—not too dim to prevent the observer seeing the pupil—to cover up one eye entirely, to hold a lighted match at the side of his head, and then to bring it forward deliberately in front of the uncovered eye.

If the pupil reacts to light, well and good. If, however, it does not, there is something the matter. If the effects of atropine, homatropine, eserine, and other similar drugs can be excluded, and also local disease that might fix the pupil, then there is some lesion in the reflex arc.

It will be remembered that, owing to the partial decussation of the optic nerves at the chiasma, some of the nerve fibres from each eye pass into the opposite optic tract as well as some into the optic tract of the same side. The right eye, for instance, is connected with the nucleus of the right third nerve by the non-decussated fibres of the right optic nerve, whilst it is also connected with the nucleus of the left third nerve by those fibres of the right optic nerve which decussate and pass up the left optic tract. Hence afferent impulses ascending from the retina of the right eye cause efferent impulses down both right and left third nerves. Therefore, if light is thrown into the right eye, not only will the right pupil contract, but the left will do so also, even though the left eye is kept in dimness.

If the right pupil fails to contract when a ray of light is thrown directly into it, but still actively contracts when a ray of light is thrown into the left eye, the block is obviously in the sensory side of the right reflex arc, its motor side, including the reflex centre, being intact and active.

If, on the other hand, the right pupil fails to contract either when a beam of light is thrown into it or when a light is thrown into the left eye, whilst the left pupil contracts both when a beam of light is thrown into the right eye and when a similar beam is thrown into the left eye, it is obvious that the block is on the motor side of the reflex arc of the right eye—that is to say, either in the region of the nucleus of the right third nerve or in the right third nerve itself, and that the sensory side of the right eye and both the motor and the sensory sides of the reflex of the left eye are intact.

(*To be concluded.*)

